# Supplementary material for: Attenuated beta rebound to proprioceptive afferent feedback in Parkinson’s disease
Source: Sci Rep. 2019 Feb 22;9:2604. doi: 10.1038/s41598-019-39204-3 (PMC6385616; doi:10.1038/s41598-019-39204-3)
Supplement: Supplementary file 1 — Supplementary material [file 41598_2019_39204_MOESM1_ESM.docx]

Supplementary material: Attenuated beta rebound to proprioceptive afferent feedback in Parkinson’s disease

**Authors**:

Mikkel C. Vinding^1^*, Panagiota Tsitsi^2^, Harri Piitulainen^3^, Josefine Waldthaler^2^, Veikko Jousmäki^1,3,4^, Martin Ingvar^1^, Per Svenningsson^2^ & Daniel Lundqvist^1^

**Affiliations**:

1) NatMEG, Department of Clinical Neuroscience, Karolinska Institutet, Stockholm, Sweden.

2) Section of Neurology, Department of Clinical Neuroscience, Karolinska Institutet, Stockholm, Sweden

3) Aalto NeuroImaging, Department of Neuroscience and Biomedical Engineering, Aalto University School of Science, Espoo, Finland

4) Cognitive Neuroimaging Centre, Nanyang Technological University, Singapore

***Corresponding author**

Mikkel C. Vinding, Ph.D.

NatMEG, Department of Clinical Neuroscience
Karolinska Institutet
Nobels väg 9, D3
171 77 Stockholm
Sweden

Email: [mikkel.vinding@ki.se](mailto:mikkel.vinding@ki.se)

# Individual beta temporal-spectral evolution


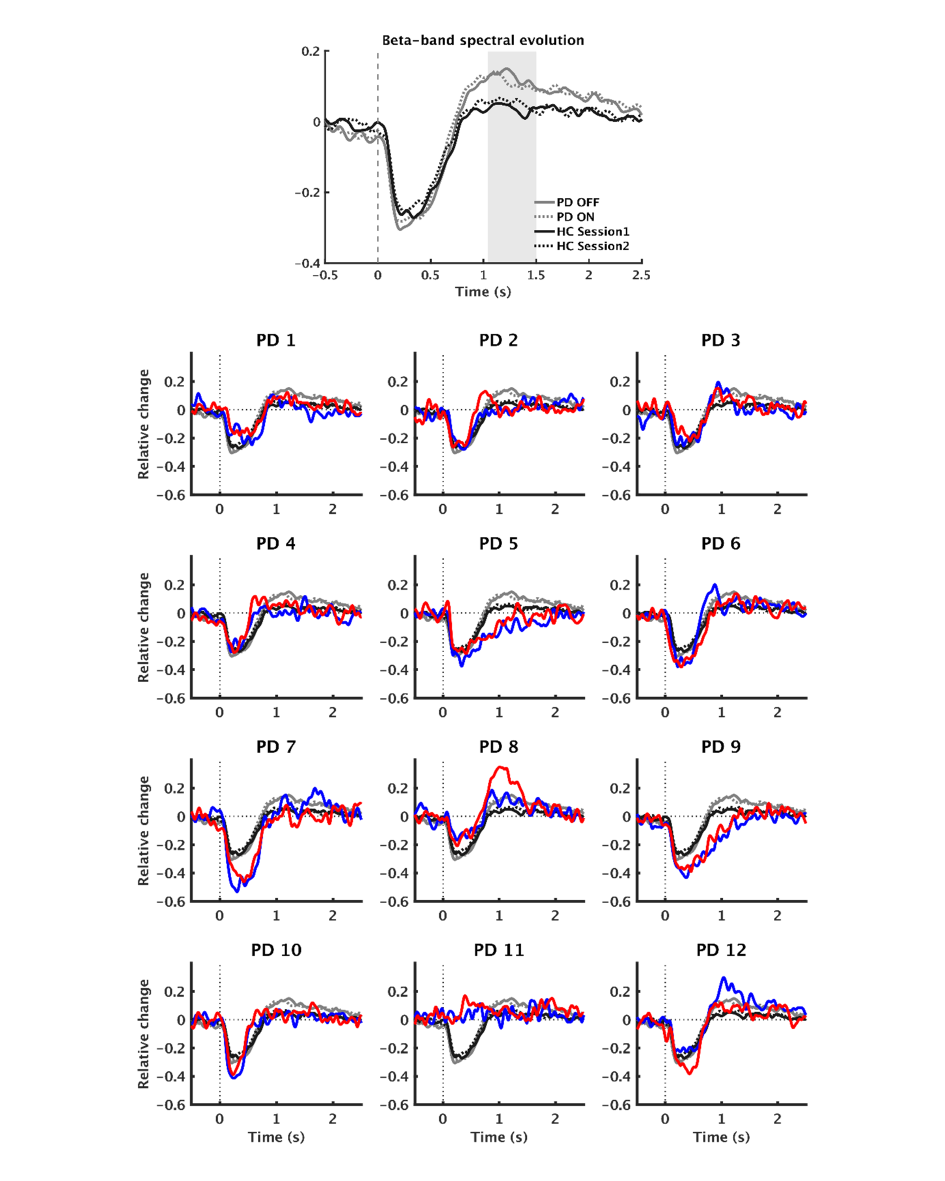


**Supplementary Figure 1**: Temporal-spectral evolutions (average across trials) in the frequency range 14-25 Hz. The top black-white figure shows the average across groups and session, with the time of the cluster that showed a difference between groups. The red and blue lines in the figures below show the response for each PD patient OFF medication (blue) and ON medication (red). The grey lines beneath the colored lines show the average temporal-spectral evolution identical to the top figure. The dark grey lines show the average response OFF medication (solid line) and ON medication (dashed line) for all PD patients. The light grey lines show the average response for all HC in the first session (solid line) and second session (dashed line).


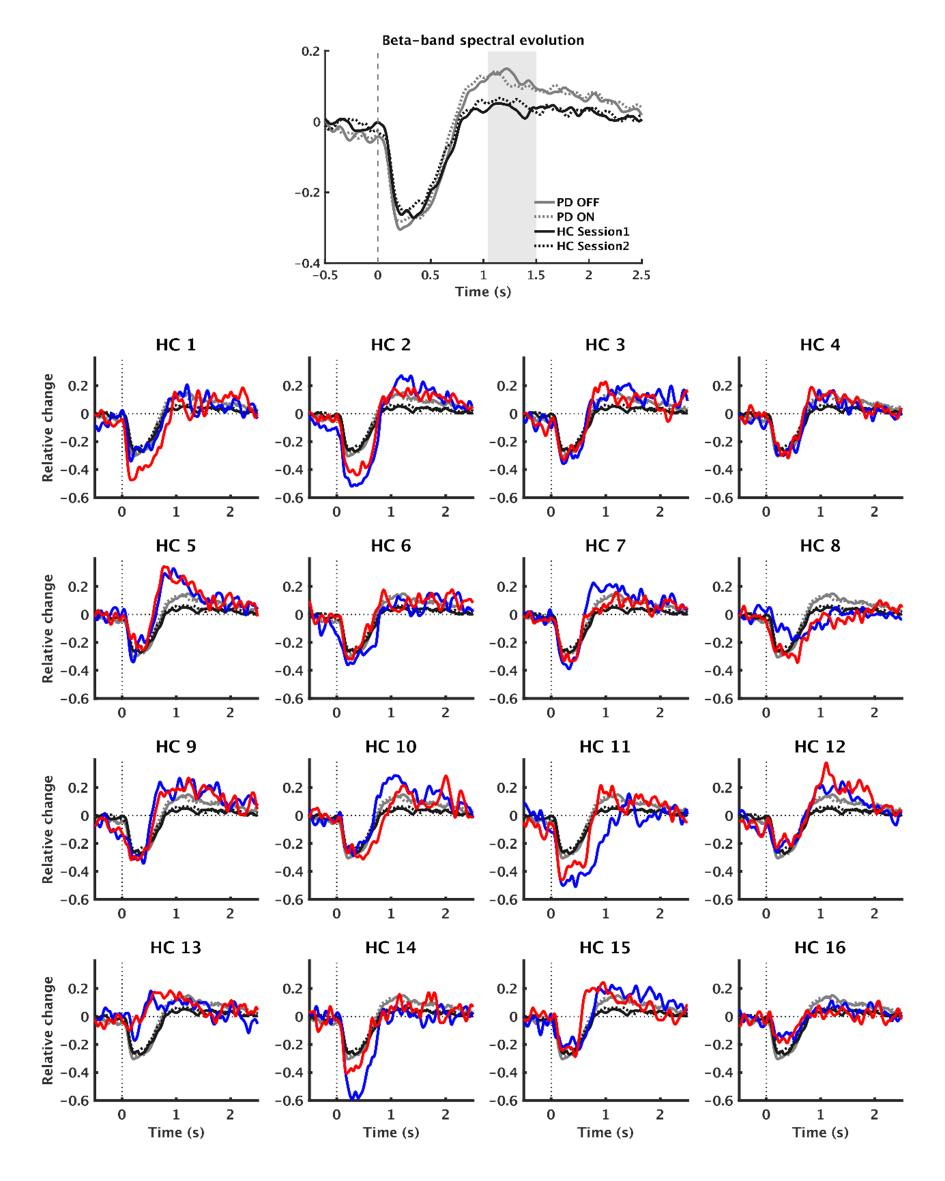


**Supplementary Figure 2**: Temporal-spectral evolutions (average across trials) in the frequency range 14-25 Hz. The top black-white figure shows the average across groups and session, with the time of the cluster that showed a difference between groups. The red and blue lines in the figures below show the response for each HC in the first session (blue) and the second session (red). The grey lines beneath the colored lines show the average temporal-spectral evolution identical to the top figure. The dark grey lines show the average response of PD patients OFF medication (solid line) and ON medication (dashed line). The light grey lines show the average response for all HC in the first session (solid line) and second session (dashed line).

# Correlation between beta rebound change and MDS-UPDRS-III scores

Due to the relatively small sample size of PD patients (n=12), we have not included an analysis of the correlation between beta-rebound change and MDS-UPDRS-III scores in the main article as the correlation analysis is too underpowered. We have included the analysis here as supplementary material, so the outcome of the analysis can serve as an inspiration to generate future hypotheses.

We sought for a relationship between the time-frequency responses in the beta-band and clinical motor symptoms in PD—measured by the MDS-UPDRS-III—by extracting the mean spectra power change in clusters that informed the significant difference between groups in the main analysis. The mean spectra power change within the cluster was modeled in a linear regression model as a function of MDS-UPDRS-III scores, with random effects indicating the session (ON/OFF medication), and individual intercepts for each patient. The MDS-UPDRS-III scores were divided into seven subscales ^1^. We made separate regression models for the score of each subscale of the MDS-UPDRS-III. Each model was compared to a “null-model” containing only the random effects by Bayesian model comparison ^2^.

Bayes factor (BF) was obtained by comparing the models to a “null-model” containing only the random effects. A summary of the comparison is shown in Supplementary Table 1. Only the factor representing tremor at rest showed substantial evidence in favor of a relation between clinical score and change beta rebound. The evidence for the other subscales were all inconclusive (within the range 1/3-3), giving neither substantial support for H_1_ nor H_0_. The inconclusive test is not surprising given the small sample size.

**Supplementary Table 1**: Comparison of relative change in power of the beta rebound and different factors of the motor examination of MDS-UPDRS-III ^1^. The BF represents the evidence in favor of the model containing the factors (H1) over the model without the factor (H0). Intercept and slope are the coefficients of the regression line drawn from the posterior distribution of the model (n=2000). A positive sign of the slope indicates that a higher clinical score is associated with increased post-movement rebound. Supplementary Figure 3 gives a visual representation of the linear regression models.

| **MDS-UPDRS-III Factor** | **BF (H_1_/H_0_)** | **Intercept [95%CI]** | **slope [95%CI]** |
| --- | --- | --- | --- |
| F1: Midline function | 0.70 | 0.040 [-0.032:0.109] | 0.000 [-0.005 0.006] |
| F2: Rest tremor | 4.90* | 0.041 [-0.052:0.129] | 0.008 [-0.000:0.017] |
| F3: Rigidity | 0.95 | 0.039 [-0.035:0.109] | -0.004 [-0.017:0.005] |
| F4: Bradykinesia right^1^ | 0.85 | 0.040 [-0.054:0.136] | 0.002 [-0.006:0.014] |
| F5: Bradykinesia left^1^ | 0.75 | 0.043 [-0.034:0.127] | -0.002 [-0.014:0.007] |
| F6: Postural and kinetic tremor | 0.70 | 0.040 [-0.037:0.120] | 0.001 [-0.008:0.011] |
| F7: Lower limb bradykinesia | 0.99 | 0.041 [-0.053:0.126] | 0.004 [-0.004:0.016] |
| Total MDS-UPDRS-III score | 0.55 | 0.043 [-0.083:0.197] | 0.001 [-0.001:0.004] |

^1^ Factors switched for PD patients who were left-side dominant to make the PD dominant side in the same factor for all patients.


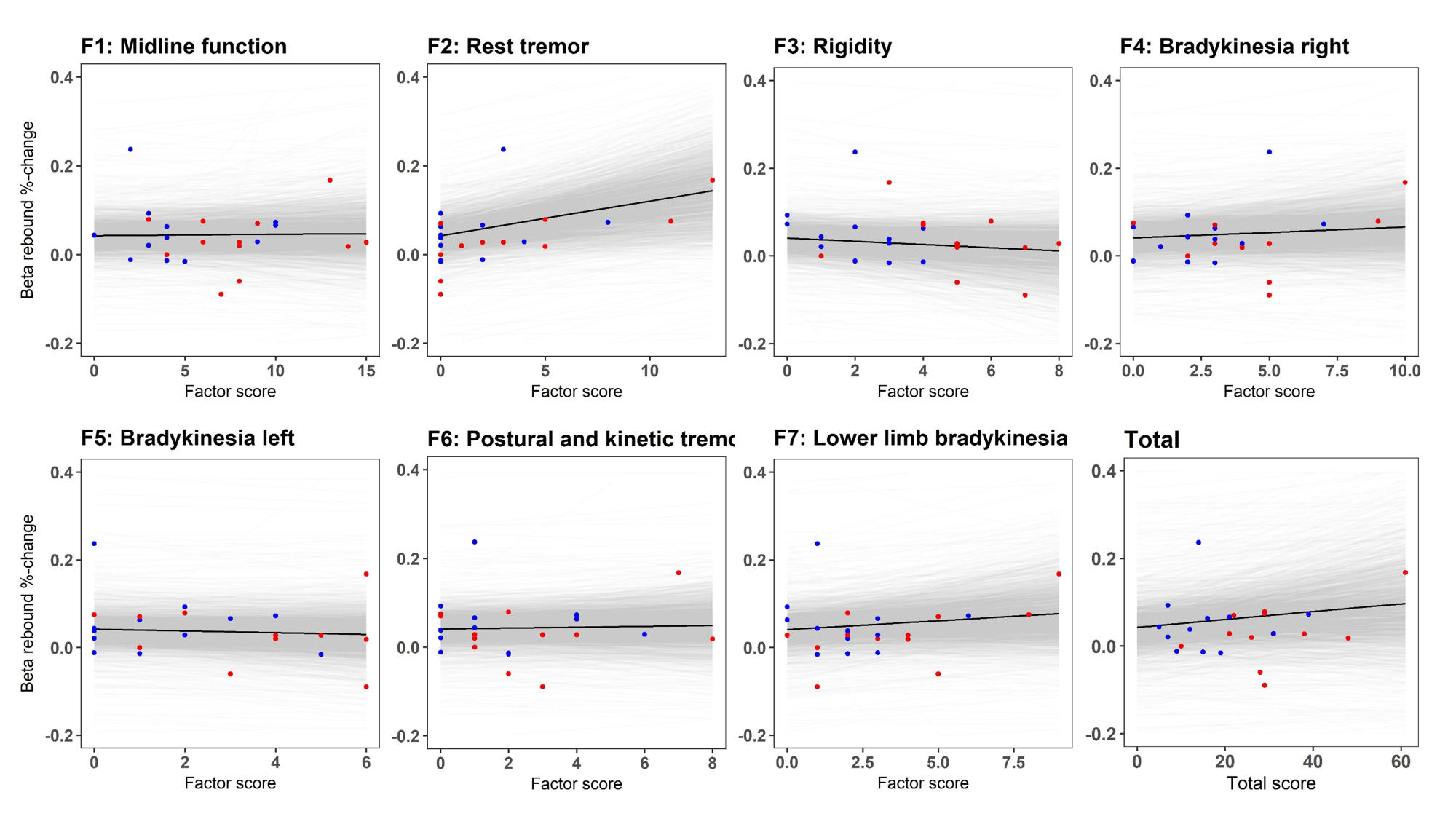


**Supplementary Figure 3:** Comparison of relative change in power of the beta rebound and different factors of the motor examination of MDS-UPDRS-III. Each dot represents the MDS-UPDRS-III score and relative change during the beta-rebound relative to the baseline. Blue dots are patients ON medication, and red dots are OFF medication. The solid lines show the mean linear regression indicating the relation between the MDS-UPDRS-III score and the beta rebound. The shade indicates the uncertainty in the regression-line: each is a thin grey line corresponding to a single sample drawn from the posterior distribution of the fitted regression model.

## References

1. Goetz, C. G. *et al.* Movement Disorder Society-sponsored revision of the Unified Parkinson’s Disease Rating Scale (MDS-UPDRS): Scale presentation and clinimetric testing results. *Mov. Disord.* **23**, 2129–2170 (2008).

2. Rouder, J. N., Morey, R. D., Speckman, P. L. & Province, J. M. Default Bayes factors for ANOVA designs. *J. Math. Psychol.* **56**, 356–374 (2012).
